# Impact of COVID-19 on Characteristics and Funding of U.S. Healthcare Startups: Retrospective Review

**DOI:** 10.2196/56327

**Published:** 2024-08-27

**Authors:** Smitha Ganeshan, Joshua Goldstein, Young-Jin Sohn, Amie Pollack, Russell S Phillips, Lisa Rotenstein

**Affiliations:** 1 Division of Hospital Medicine Department of Medicine University of California San Francisco San Francisco, CA United States; 2 Tufts University School of Medicine Boston, MA United States; 3 Center for Primary Care Harvard Medical School Boston, MA United States; 4 Division of Clinical Informatics and Digital Transformation University of California San Francisco San Francisco, CA United States; 5 Division of General Internal Medicine University of California San Francisco San Francisco, CA United States

**Keywords:** artificial intelligence, venture capital, COVID-19, health care startup, health care, AI, retrospective study, telehealth, telemedicine, pandemic, patients, digital health, coronavirus

## Abstract

**Background:**

The rise of telehealth and telemedicine during the pandemic allowed patients and providers to develop a sense of comfort with telehealth, which may have increased the demand for virtual-first care solutions with spillover effects into venture capital funding.

**Objective:**

We aimed to understand the size and type of digital health investments occurring in the prepandemic and pandemic periods.

**Methods:**

We examined health care companies founded from March 14, 2019, to March 14, 2020 (prepandemic) versus those founded from March 15, 2020, to March 14, 2022, after pandemic onset. Data were obtained from Crunchbase, a publicly available database that catalogs information about venture capital investments for companies. We also compared companies founded prepandemic to those founded after the first year of the pandemic (pandemic steady-state). We performed a Wilcoxon rank sum test to compare median funding amounts. We compared the 2 groups of companies according to the type of funding round raised, geography, health care subcategory, total amount of funding per year since founding, and number of founders.

**Results:**

There were 2714 and 2218 companies founded prepandemic and during the pandemic, respectively. The companies were similarly distributed across geographies in the prepandemic and pandemic periods (*P*=.46) with no significant differences in the number of founders (*P*=.32). There was a significant difference in total funding per year since founding between prepandemic and pandemic companies (US $10.8 million vs US $20.9 million; *P*<.001). The distribution of funding rounds differed significantly for companies founded in prepandemic and pandemic periods (*P*<.001). On excluding data from the first year of the pandemic, there were 581 companies founded in the pandemic steady-state period from March 14, 2021, to March 14, 2022. Companies founded prepandemic had a significantly greater mean number of founders than those founded during the pandemic (*P*=.02). There was no significant difference in total funding per year since founding between prepandemic and steady-state pandemic companies (US $10.8 million vs US $14.4 million; *P*=.34). The most common types of health care companies included wellness, biotech/biopharma, and software companies. Distributions of companies across health care subcategories were not significantly different before and during the pandemic. However, significant differences were identified when data from the first year of the pandemic were excluded (*P*<.001). Companies founded during the steady-state pandemic period were significantly more likely to be classified as artificial intelligence (7.3% vs 4.7%; *P*=.005), software (17.3% vs 12.7%; *P*=.002), and insurance (3.3% vs 1.7%; *P*=.003), and were significantly less likely to be classified as health care diagnostics (2.4% vs 5.1%; *P*=.002).

**Conclusions:**

We demonstrate no significant changes in the types of health care companies founded before versus during the pandemic, although significant differences emerge when comparing prepandemic companies to those founded after the first year of the pandemic.

## Introduction

The COVID-19 pandemic rapidly shifted priorities for health care delivery. Patients and providers gained comfort with telehealth [1] and increasingly relied on this modality of care delivery [2]. Pressures related to social distancing created an increased focus on home-based care and new diagnostic methods to test for COVID-19, which may have impacted the types of companies founded [2-6].

Unlike stock markets, venture capital is less impacted by individual investors and can assess the reallocation of capital in response to major events [7]. Industry and published research have shown significant venture activity in health-related areas of healthtech, biopharma, devices, and diagnostic tools; however, there has been relatively less exploration on changes in investments between prepandemic and pandemic periods [8-11]. One study on the impact of COVID-19 on venture investments found that venture capitalists invested up to 44% more capital in pandemic-related fields. Another study found that half of venture capitalists reported a positive impact of COVID-19 on investments. However, another white paper found that early stage venture capitalist activity declined by 38% [12,13].

Although these studies inform our understanding, little data exist on the impact of the COVID-19 pandemic on the specific types of health care investments in the United States where overall venture spending has increased [14]. Our objective was to understand the types of health care companies founded before versus after the COVID-19 pandemic.

**Figure 1:**
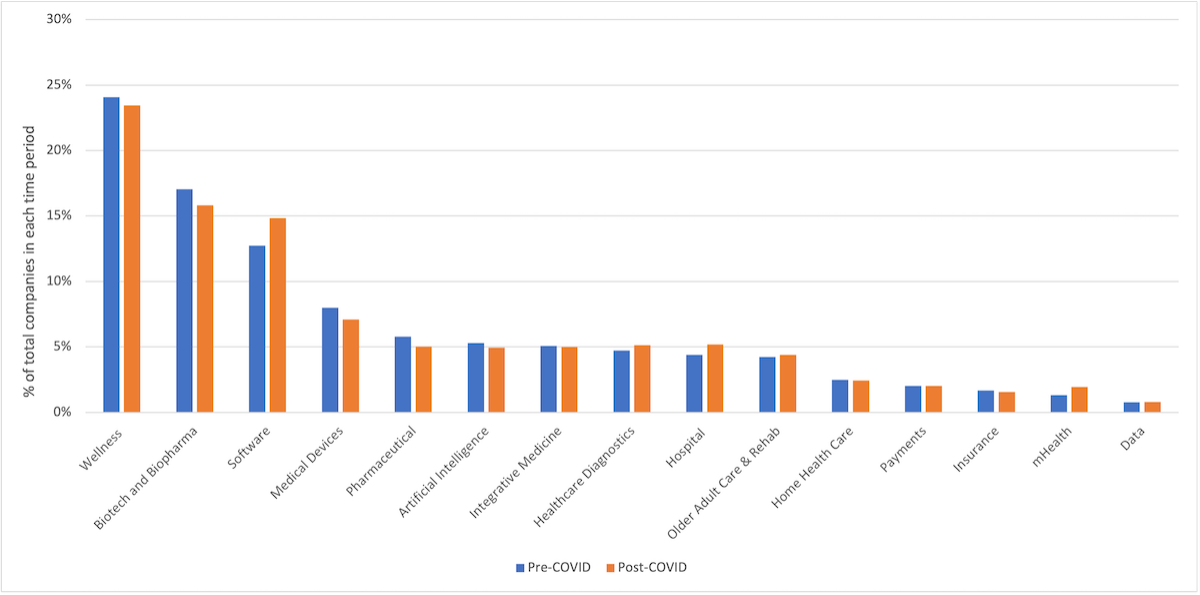
Categorizations of venture-backed healthcare companies pre- and post-pandemic.

## Methods

### Data Source

We obtained data on US health care companies from Crunchbase, a web-based database cataloging startup information about company variables and health care subcategories from users, public data, and other data partners. Companies and users self-classify into preexisting subcategory tags and can use multiple similar or overlapping tags. We investigated US companies founded from March 14, 2019, to March 14, 2022.

### Outcomes

We extracted data on geography, profit structure, funding round, amount of total funding, and number of founders. We grouped Crunchbase’s health care subcategories into broader groupings (see Multimedia Appendix 1). Using the companies’ total funding to date and founding dates, we calculated the total funding per year for comparison across companies.

### Analysis Approach

In our primary analysis, we compared the characteristics of US health care companies founded from March 14, 2019, to March 14, 2020 (prepandemic period) to those founded from March 15, 2020, to March 14, 2022 (pandemic period). In a secondary analysis, we compared the characteristics of companies founded prepandemic to those founded from March 14, 2021, to March 14, 2022, when the initial pandemic peak subsided (pandemic steady-state period).

We used descriptive statistics to describe the characteristics of companies founded in the prepandemic, during pandemic, and steady-state pandemic periods. We used chi-square tests to compare categorical variables and Wilcoxon rank sum tests to compare continuous variables. *P*<.05 was used to assess statistical significance.

### Ethical Considerations

As this study used publicly available data and did not involve human subjects, institutional review board review was not required. We have adhered to local, national, regional, and international law and regulations regarding the protection of personal information, privacy, and human rights [15].

## Results

According to Crunchbase, there were 2714 companies founded prepandemic and 2218 companies founded after the start of the pandemic (characteristics summarized in Table 1).

The mean funding per year by health care category did not significantly differ prepandemic and during the pandemic (Table 2).

When data from the first year of the pandemic were excluded, there were 581 companies founded in the pandemic steady-state period (Table 3). Data on geographic distribution, funding rounds, and number of founders were similar between time points. However, in contrast to analyses including the period from March 15, 2020, to March 14, 2021, in analyses comparing the prepandemic period to the pandemic steady-state period, there was no significant difference in the total funding per year since founding (Table 3).

The most common types of health care companies both prepandemic and during the pandemic included wellness (24.1% prepandemic and 23.4% during the pandemic), biotech/biopharma (17.0% prepandemic and 15.8% during the pandemic), and software (12.7% prepandemic and 14.8% during the pandemic). Data companies (0.7% prepandemic and 0.8% during the pandemic) and fertility companies (0.3% prepandemic and 0.3% during the pandemic) had a lower proportion of investments. The distributions of companies across health care subcategories were not significantly different before versus during the pandemic.

However, when data from the first year of the pandemic were excluded, there were significant differences. Companies founded during the pandemic were significantly more likely to be classified as artificial intelligence (7.3% in the pandemic steady-state vs 4.7% prepandemic; *P*=.005), software (17.3% in the pandemic steady-state vs 12.7% prepandemic; *P*=.002), and insurance (3.3% in the pandemic steady-state vs 1.7% prepandemic; *P*=.003), and significantly less likely to be classified as health care diagnostics (2.4% in the pandemic steady-state vs 5.1% prepandemic; *P*=.002).

**Table 1:** Characteristics of companies founded prepandemic and during the pandemic.

Characteristic			Prepandemic: March 15, 2019, to March 14, 2020 (n=2714)		During the pandemic: March 15, 2020, to March 14, 2022 (n=2218)		*P* value	
**Geography, n (%)**								.46
	Northeast	697 (25.68)		526 (23.72)				
	Midwest	300 (11.05)		248 (11.18)				
	South	770 (28.37)		650 (29.30)				
	West	947 (34.89)		794 (35.80)				
**Funding round, n (%)^a^**								<.001
	Seed	586 (69)		618 (80)				
	Early stage	173 (20)		113 (15)				
	Private equity	11 (1.3)		8 (1.0)				
	IPO^b^	16 (1.9)		14 (1.8)				
	M&A^c^	57 (6.7)		17 (2.2)				
	Late stage	8 (0.9)		1 (0.1)				
Total funding per year since founding (US $), mean (SD)^d^			10,832,252 (45,669,417)		20,970,037 (119,295,457)		<.001	
Number of founders, mean (SD)^e^			1.75 (0.96)		1.72 (0.93)		.32	

^a^Data available for 851 companies prepandemic and 771 companies during the pandemic.

^b^IPO: initial public offering.

^c^M&A: mergers and acquisitions.

^d^Data available for 901 companies prepandemic and 759 companies during the pandemic.

^e^Data available for 1417 companies prepandemic and 1093 companies during the pandemic.

**Table 2:** Median total funding amount per year by health care category.

Health care category	Prepandemic: March 14, 2019, to March 14, 2020 (n=2714)		During the pandemic: March 15, 2020, to March 14, 2022 (n=2218)			*P* value
	Median total funding per year (US $)	Companies with available data, n (%)	Median total funding per year (US $)	Companies with available data, n (%)		
Wellness	2,000,000	219/749 (29.2)	1,311,250	184/588 (31.3)	.27	
Integrative medicine	1,750,000	51/165 (30.9)	1,556,000	36/124 (29.0)	.91	
Care for older adults and rehabilitation	1,400,000	19/132 (14.4)	1,000,000	21/110 (19.1)	.66	
Mobile health	1,000,000	17/41 (41.5)	1,500,000	21/49 (42.9)	>.99	
Artificial intelligence	1,425,000	73/147 (49.7)	1,000,000	62/129 (48.1)	.60	
Software	2,000,000	172/396 (43.4)	1,000,000	147/372 (39.5)	.12	
Payments	10,050,000	26/77 (33.8)	3,500,000	25/61 (41.0)	.19	
Insurance	2,436,500	18/52 (34.6)	1,500,000	19/39 (48.7)	.73	
Data	11,000,000	9/24 (37.5)	800,000	12/20 (60.0)	.29	
Pharmaceutical	6,684,975.50	80/180 (44.4)	4,812,500	48/126 (38.1)	.88	
Fertility	1,485,000	6/10 (60.0)	2,803,500	5/9 (55.6)	.72	
Health care diagnostics	3,000,000	53/158 (33.5)	2,116,750	50/125 (40.0)	.98	
Biotech and biopharma	6,000,000	265/530 (50.0)	10,000,000	195/397 (49.1)	.14	
Health systems	3,271,673	26/137 (19.0)	2,200,000	31/130 (23.8)	.86	
Medical device	1,370,000	105/249 (42.2)	1,440,000	87/178 (48.9)	.63	
Home health care	2,900,000	18/63 (28.6)	630,000	15/51 (29.4)	.29	

**Table 3:** Characteristics of companies founded prepandemic and during the pandemic steady-state.

Characteristic		Prepandemic: March 14, 2019, to March 14, 2020 (n=2714)	Pandemic steady-state: March 14, 2021, to March 14, 2022 (n=581)	*P* value	
**Geography, n (%)**				.71	
	Northeast	697 (25.68)	136 (23.41)		
	Midwest	300 (11.05)	64 (11.02)		
	South	770 (28.37)	171 (29.43)		
	West	947 (34.89)	210 (36.14)		
**Funding round, n (%)^a^**					<.001
	Seed	586 (69)	200 (86.6)		
	Early stage	173 (20)	26 (11.3)		
	Private equity	11 (1.3)	0 (0)		
	IPO^b^	16 (1.9)	2 (0.9)		
	M&A^c^	57 (6.7)	3 (1.3)		
	Late stage	8 (0.9)	0 (0)		
Total funding per year since founding (US $), mean (SD)^d^		10,832,252 (45,669,417)	14,348,739 (52,119,919)	.34	
Number of founders, mean (SD)^e^		1.75 (0.96)	1.62 (0.88)	.02	

^a^Data available for 851 companies prepandemic and 231 companies in the pandemic stead state.

^b^IPO: initial public offering.

^c^M&A: mergers and acquisitions.

^d^Data available for 901 companies prepandemic and 201 companies in the pandemic steady-state.

^e^Data available for 1417 companies in prepandemic and 312 in the pandemic steady-state.

## Discussion

In this national cross-sectional study of startup companies, we demonstrate significant differences in total funding per year since founding of US $10.8 million per year prepandemic compared to US $20.9 million per year during the pandemic. We also demonstrate a significant increase in the proportion of companies in the seed stage during the pandemic. Our results did not reveal significant differences in the types of companies founded prepandemic and during the first 2 years of the pandemic. However, we found a significant difference in the types of companies founded prepandemic versus during the pandemic steady-state, with a 55.3% relative increase in the proportion of companies classified as dealing with artificial intelligence, a 36.2% relative increase in the proportion of companies classified as software, and a 52.9% relative decrease in the proportion of companies labeled as health care diagnostics. Overall, wellness, biotech/biopharma, and software companies accounted for the highest proportions of founded companies overall, with relatively less activity in the fertility category.

The significant increase in total funding per year prepandemic compared to that during the pandemic is supported by existing data demonstrating an increase in global venture funding from 2020 to 2022 compared to 2019 [16]. This may suggest that the pandemic spurred increased activity in innovation; however, to our knowledge, no research to date has compared differences in venture capital investments specifically before and after the start of the pandemic [17,18].

Our results did not reveal significant differences in the types of companies founded prepandemic and during the first 2 years of the pandemic. Given the significant lead time needed for founders to move from idea generation to founding a company and raising money, we hypothesized that the first year of the pandemic may have been more reflective of prepandemic trends and may not have captured shifts in the market from the pandemic itself. When we compared prepandemic companies to those founded during the pandemic steady-state, we did find significant increases in artificial intelligence, software, and biotech investments, although this may also be influenced by longer-standing market trends [18,19]. While we did not find a significant increase in the proportion of mobile health (mHealth) companies, despite the rise of virtual care during the pandemic, this expected trend may have been captured by the increase in the proportion of software companies, which includes many mHealth companies. Industry research supports our finding of high levels of venture investment in the areas of artificial intelligence, biotech, and software/digital health/healthtech [20-22].

The significant relative decrease in health care diagnostic companies despite the increase of at-home COVID-19 testing is surprising. It is possible that diagnostics were largely being developed by larger, traditional companies rather than newer startups. It is also possible that the pandemic period coincided with a higher inflationary environment that made investors and founders more conservative in more capital-intensive areas such as diagnostics [23-25]. A study published in 2020 from the National Bureau of Economic Research found that from 1974 to 2019, during economic downturns, venture capital firms changed their investment focus toward less innovative startups [13].

Some Crunchbase data are directly derived from site users, which may contribute to selective reporting bias. Further, there are no standard definitions of health care subcategories for users to base their categorizations on. Companies can be tagged to multiple relevant health care categories, which may overcome some ambiguity in definition. Nevertheless, Crunchbase represents one of the only publicly available repositories of startup company data, and our study is the first to leverage these data to understand the trends in the founding of health care companies.

In conclusion, we demonstrate no changes in the distribution of focus areas for companies founded before after the start of the COVID-19 pandemic; however, when we isolate pandemic steady-state data, we see significantly increased activity related to the fields of artificial intelligence and software and significantly less activity in health care diagnostics. This may reflect the impacts of the COVID-19 pandemic on investing patterns. As health care venture capital investments more actively shape the health care delivery landscape, real-time efforts to aggregate information on company establishment and venture capital investments would allow health system researchers to better understand innovation trends and the flow of capital in health care.

## Appendix

Multimedia Appendix 1 Grouping of Crunchbase health care categories into broader categories.

## Abbreviations

mHealth: mobile health

## Data Availability

The data sets analyzed during this study are not publicly available due to being proprietary data from Crunchbase, but are available from the corresponding author on reasonable request.
