# Supplementary material for: Impact of COVID-19 on Characteristics and Funding of U.S. Healthcare Startups: Retrospective Review
Source: JMIR Form Res. 2024 Aug 27;8:e56327. doi: 10.2196/56327 (PMC11387907; doi:10.2196/56327)
Supplement: Multimedia Appendix 1 [file formative_v8i1e56327_app1.docx]

*Appendix 1. Grouping of Crunchbase healthcare categories into broader categories*

| **Name** | **Tags** |
| --- | --- |
| Wellness | Wellness, Personal Health, Nutrition, |
| Integrative Medicine | alternative medicine / cannabis / dietary supplements / Nutraceutical |
| Care for older adults and rehabilitation | assisted living / assistive technology / elder care / nursing and residential care / rehabilitation |
| Mobile Health (mHealth) | mHealth |
| Artificial Intelligence | artificial intelligence / machine learning / natural language processing |
| Software | Information Services /Information Technology /Information and Communications /Management Information Systems /Software Engineering / Software / Computer /Consumer Software /Developer APIs /Wireless / electronic health record (ehr) |
| Payments | Cryptocurrency / Payments / Personal Finance / Fintech / Finance / Financial Services |
| Insurance | Health Insurance / Insurance / InsurTech |
| Data | Big Data / Bioinformatics / Biometrics / Data and Analytics |
| Pharmaceutical | clinical trials / pharmaceutical |
| Fertility | Fertility |
| Healthcare Diagnostics | health diagnostics |
| Biotech and Biopharma | biotechnology/ biopharma / therapeutics |
| Health systems | emergency medicine / hospital |
| Medical Device | medical device/wearables / Hardware |
| Home Health Care | Home health care |
